# Supplementary material for: Scalable reinforcement learning for large-scale coordination of electric vehicles using graph neural networks
Source: Commun Eng. 2025 Jul 1;4:118. doi: 10.1038/s44172-025-00457-8 (PMC12216737; doi:10.1038/s44172-025-00457-8)
Supplement: Supplementary file 2 — Supplementary Information [file 44172_2025_457_MOESM2_ESM.pdf]

Supplementary Material for: Scalable Reinforcement Learning for Large-Scale Coordination of Electric Vehicles Using Graph Neural Networks

Stavros Orfanoudakis, Valentin Robu, E. Mauricio Salazar,
Peter Palensky, Pedro P. Vergara

1 Heuristic Algorithms for EV charging

In this section implementation details of two most used heuristic algorithms for EV charging are presented.

1.1 Charge As Fast As Possible (AFAP)

As the name implies, the Charge As Fast As Possible (AFAP) algorithm continuously supplies the maximum possible charging power to EVs without accounting for operational constraints. In simulation, this can be represented using a Markov Decision Process (MDP), where the chosen action is consistently set to the highest possible value (in this case, 1).

1.2 Round Robin (RR)

The Round Robin (RR) algorithm for EV charging distributes power in a cyclic, fair manner among all connected EVs, ensuring each receives a fair share of the available charging capacity. Unlike approaches prioritizing maximum power delivery, RR balances power allocation by assigning a portion of power to each EV sequentially, looping through the queue repeatedly until the total power limit is reached. The process, as outlined in Algorithm 1, simulates a scheduling mechanism where each EV is allocated a share of power in succession, maintaining a balanced distribution within the operational constraints.

Algorithm 1 Round Robin for EV Charging

Input: Set of EVs $E = \{EV_0, EV_1, \ldots, EV_n\}$, each EV_i with minimum charging power $\underline{P}_i$ and a maximum charging power $\overline{P}_i$, and an aggregated total power limit P^{set}.
$P^{\text{tot}} = 0$
$i = 0$
while $P^{\text{tot}} < P^{\text{set}}$ **do**
  if $P^{\text{tot}} + \underline{P}_i \leq P^{\text{set}}$ **then**
    Assign $\underline{P}_i$ to EV_i
    $P^{\text{tot}} \leftarrow P^{\text{tot}} + \underline{P}_i$
  else
    Assign remaining power $P^{\text{set}} - P^{\text{tot}}$ to EV_i
    $P^{\text{tot}} \leftarrow P^{\text{set}}$
  end if
  Increment $i \leftarrow i + 1 \pmod{n}$ to the next EV
end while

2 Additional Results

This section provides supplementary training results.

2.1 RL Training Performance Comparison

Supplementary Fig. 1 provides an in-depth comparison of the training performance for SAC and TD3, the top-performing classic RL algorithms, along with their variations incorporating FX-GNN and EV-GNN. Fig. 1 illustrates the maximum reward achieved after five training runs, highlighting the number of epochs required to achieve it. The sample efficiency of the proposed approach is clearly demonstrated in the 25 CP case, where EV-GNN reaches its maximum reward in approximately 500 epochs, compared to 2000 epochs for FX-GNN and 3500 epochs for the classic approaches. This trend continues in the 100 CPs scenario, where EV-GNN achieves its maximum reward in nearly half the number of epochs required by FX-GNN. Classic SAC and TD3, however, exhibit significant scalability issues, even with just 100 CPs, and these limitations become increasingly apparent in the 500 and 1000 CP cases. In contrast, EV-GNN consistently learns and achieves the highest rewards in large-scale experiments, showing substantial improvements over both FX-GNN and the classic approaches.

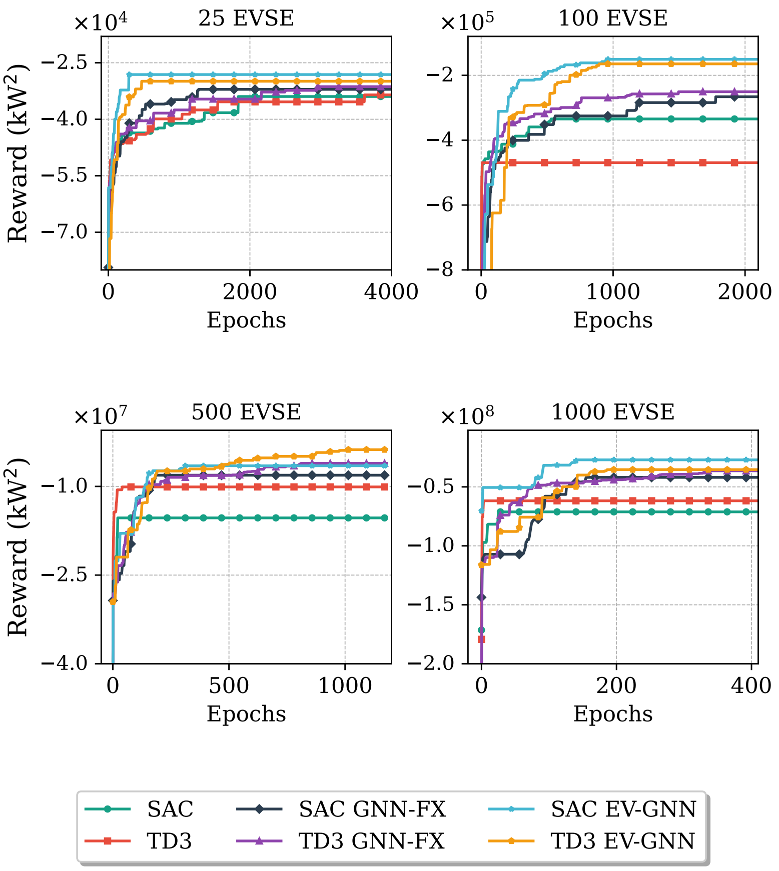

Supplementary Figure 1 Maximum reward comparison across different experiment scales. This graph illustrates the maximum rewards achieved by different approaches after 5 training runs with different random seeds. The methods compared include the end-to-end EV-GNN approach, the GNN feature extractor (FX-GNN), and the classic versions of SAC and TD3. Lower CP numbers (e.g., 25) indicate simpler tasks with a 25-dimensional action space and a 75-dimensional state space, whereas the larger 1000 CP case represents a highly complex optimization task with a 1000-dimensional action space and a state space exceeding 3000 dimensions.
